# Description of *Colponema vietnamica* sp.n. and *Acavomonas peruviana* n. gen. n. sp., Two New Alveolate Phyla (Colponemidia nom. nov. and Acavomonidia nom. nov.) and Their Contributions to Reconstructing the Ancestral State of Alveolates and Eukaryotes

**DOI:** 10.1371/journal.pone.0095467

**Published:** 2014-04-16

**Authors:** Denis V. Tikhonenkov, Jan Janouškovec, Alexander P. Mylnikov, Kirill V. Mikhailov, Timur G. Simdyanov, Vladimir V. Aleoshin, Patrick J. Keeling

**Affiliations:** 1 Canadian Institute for Advanced Research, Botany Department, University of British Columbia, Vancouver, British Columbia, Canada; 2 Institute for Biology of Inland Waters, Russian Academy of Sciences, Borok, Yaroslavl Provence, Russia; 3 Belozersky Institute for Physicochemical Biology, Lomonosov Moscow State University, Moscow, Russia; 4 Faculty of Biology, Lomonosov Moscow State University, Moscow, Russia; Institut Pasteur, France

## Abstract

The evolutionary and ecological importance of predatory flagellates are too often overlooked. This is not only a gap in our understanding of microbial diversity, but also impacts how we interpret their better-studied relatives. A prime example of these problems is found in the alveolates. All well-studied species belong to three large clades (apicomplexans, dinoflagellates, and ciliates), but the predatory colponemid flagellates are also alveolates that are rare in nature and seldom cultured, but potentially important to our understanding of alveolate evolution. Recently we reported the first cultivation and molecular analysis of several colponemid-like organisms representing two novel clades in molecular trees. Here we provide ultrastructural analysis and formal species descriptions for both new species, *Colponema vietnamica* n. sp. and *Acavomonas peruviana* n. gen. n. sp. Morphological and feeding characteristics concur with molecular data that both species are distinct members of alveolates, with *Acavomonas* lacking the longitudinal phagocytotic groove, a defining feature of *Colponema*. Based on ultrastructure and molecular phylogenies, which both provide concrete rationale for a taxonomic reclassification of Alveolata, we establish the new phyla Colponemidia nom. nov. for the genus *Colponema* and its close relatives, and Acavomonidia nom. nov. for the genus *Acavomonas* and its close relatives. The morphological data presented here suggests that colponemids are central to our understanding of early alveolate evolution, and suggest they also retain features of the common ancestor of all eukaryotes.

## Introduction

Alveolates comprise a great portion of protist diversity and include many medically and ecologically important species, such as the malaria parasite (*Plasmodium*), toxic red-tide algae (*Alexandrium*, *Karenia*, *Pfiesteria*), and coral endosymbionts (*Symbiodinium*, *Chromera*). Because of their significance to humans and ecosystems, some alveolate species have been extensively studied, but a great many other species of smaller apparent importance have been overlooked. This is particularly the case for free-living alveolates, such as colpodellids, chromerids, and colponemids, which fall outside the three major alveolate subgroups, apicomplexans, dinoflagellates and ciliates. However, the intermediate evolutionary positions occupied by these organisms make them particularly important for elucidating the origin and evolution of their better-studied alveolate relatives. This has recently been exemplified by *Chromera velia*: the origin of apicomplexans has been a long-standing question of interest, in particular because they have been found to have a plastid [1], [2], despite being a phylum of obligate parasites. The key to their ancestry was provided only recently through the discovery of *C. velia* because it is a close free-living sister to apicomplexans, but retains the ancestral state of photosynthesis [3]. Only by studying the *C. velia* plastid genome was the common ancestry of apicomplexan and dinoflagellate plastids made clear, because of its shared similarities to both major groups [4]. Several other alveolates, such as *Acrocoelus*, colpodellids, colponemids, *Oxyrrhis*, *Parvilucifera*, *Perkinsus*, *Rastrimonas*, and *Vitrella* similarly do not branch within any of the major alveolate subgroups, and each offers a similar evolutionary potential [5]–[23].

The colponemids, comprising the single described genus *Colponema*, also belong to this list, but deserve particular attention. Colponemids lack secondarily-derived characteristics found in other alveolates (apical cones, rhoptries, derived ciliature or palintomy), and much of their basic morphology has been proposed to have been ancestral to all alveolates [24]. For example, the obligate eukaryovory of colponemids has been established based on their feeding habits in both natural samples and temporary laboratory cultures, and is clearly supported by their longitudinal groove ultrastructure (microtubule band armoring) and presence of extrusomes (toxicysts) [25], [26]. The extrusomes are interspersed through the three-membrane pellicle resulting from presence of discrete cortical alveoli. These characteristics are also found in some ciliates, basal dinoflagellates and apicomplexans, and could have been present in their common ancestor [24]. Ancestral molecular characters in colponemids might similarly help solve the enigmatic evolutionary origins of some of the very strange genomic features of alveolates, such as the spliced leaders and polycistronic gene transcription in dinoflagellates, two distinct nuclear genomes in ciliates, origin of the apical complex structure in apicomplexans, the evolution of endosymbiotic organelles and their very unusual genomes [3], [4], [20], [27]–[35].

Despite this evolutionary promise, we known little of colponemids because of their apparently rarity in nature and the difficulty in culturing them. Colponemids remained one of the last alveolate groups for which molecular data was completely missing until recently, and only five colponemid species have been ever described, of which only two have been investigated at the ultrastructural level [7], [25], [26]. Recently, however, we reported the isolation and cultivation of two new predatory colponemid-like alveolates from Vietnam and Peru, and showed using molecular data that they represent two distinct lineages of alveolate that do not branch with any of the three major lineages [36]. Here we provide formal descriptions of these two new species, *Colponema vietnamica* sp. n. and *Acavomonas peruviana* n. gen. n. sp., including morphological and ultrastructural descriptions. Based on their ultrastructure and phylogenetic position, we conclude these genera represent two new alveolate phyla, and revise the higher level taxonomy accordingly. Analysis of their ultrastructure also suggest they retain cytoskeletal characteristics of the ancestral alveolate, and comparison with the canonical cytoskeletal features of the excavate body plan furthermore suggests some of these characteristics may be ancestral to all eukaryotes.

## Materials and Methods

### Sample Collection

New species were identified from samples collected in three distant localities: southern Vietnam wetlands (1), saline lake sediments of Peru seashore (2), and soil and permafrost of Russia (3). Vietnamese samples came from a lake and pool belonging to the Bau Sau wetland complex located in the Cát Tiên National Park, Dong Nai Province, S.R. Vietnam. Cát Tiên National Park has an area of approximately 720 km^2^ and is located approximately 150 km to the north of Ho Chi Minh City, in the south of Vietnam. The park includes a large area of lowland evergreen tropical and deciduous forest, bamboo woodlands, wetlands and seasonally flooded grasslands, and a small proportion of farmland. The territory is subjected to the tropical monsoon climate with two distinct seasons: a rainy season from April to November and a dry season from December to March. The mean annual rainfall is 2450 mm. The temperature amplitude is very low, varying from 24 to 29°C, and the mean annual temperature is 25.4°C [53]. Four clones of *Colponema* (Colp-7, Colp-7a, Colp-14, and Colp-62) were isolated. Clone Colp-7 was obtained from the sediment of the shallow boggy Dau Tron Lake (107°20′50″ E, 11°28′47″ N) on November 24, 2010. The sample was collected at 40 cm depth (Temp. 28.8°C, pH 5.66, Conductivity 12 µS/cm) and contained organic detritus, plant debris and filamentous algae. Clone Colp-7a was obtained from the sediment of the shallow pool near the forest road of National Park (107°25′55.6″ E, 11°26′38.1″ N) on May 14, 2012. The sample was collected at 20 cm depth (Temp. 27.7°C, pH 6.76, Eh 162 mV, Conductivity 115 µS/cm) and contained organic detritus, plant debris and filamentous algae. Clones Colp-14 and Colp-62 ware obtained from the sediment of the grass boggy pool close to Bau Sau Lake (107°20′21.8″ E, 11°27′15.5″) on April 28, 2013. The sample was collected at 15 cm depth (Temp. 41.12°C, pH 6.67, Eh -45.2 mV, DO 3.25 ppm, Conductivity 227 µS/cm) and contained mainly plant debris.

Peruvian samples came from sediments of the saline lake Supay (76°14′44.38″ W, 14°0′5.18 N″), Pisco Province, Ica Department, Peru. This coastal desert area is subjected to the hot arid climate. The sample was collected at 20 cm depth, (Salinity 35‰, Temp. about 25°C) and contained mainly organic detritus. One clone of the predatory flagellate Colp-5 was isolated.

Russian samples came from soil from the Vorontsovskaya cavernae system, Caucasus and from the permafrost material near Kolyma River, Chukotka, Russia as described previously [43], [54], [55]. Two clones of *Colponema edaphicum* were isolated from each region.

Field studies in Vietnam were conducted under permits issued by the administration of Cát Tiên National Park, Vietnam, and authorized by Russian-Vietnam Tropical Centre, Coastal Branch (Nha Trang, Vietnam). No specific permits were required for the described field studies in Russia and Peru. The field studies did not involve endangered or protected species.

### Culture Establishment and Maintenance

The sediment samples, including water, were placed in 50-ml flasks and transported to the laboratory within 5 days. 10 ml sample volumes (water with sediments particles) were analyzed in glass Petri dishes. Species diversity was studied directly after sample arrival and after enrichment with a suspension of *Pseudomonas fluorescens* Migula 1895 bacteria (strain ICISC19, Institute for Cellular and Intracellular Symbiosis Collection, Russian Academy of Science, Russia) as a food source. Samples were maintained in darkness at 25°C. The samples were examined on the third, sixth and ninth days of incubation in accordance with methods described previously [56], [57]. New species of predatory flagellates were observed on the sixth and ninth days.

Clonal cultures were isolated from single cells using a micromanipulator fitted with a glass micropipette [58]. Single cells were transferred to a Petri dish containing a clonal culture of a bacteriotrophic flagellate as food. The freshwater chrysomonad *Spumella* sp. was used as food for clone Colp-7 and *Colponema edaphicum*. *Spumella* sp. (strain OF-40, Institute of the Biology of Inland Waters, Russian Academy of Sciences, Russia (IBIW RAS)) was isolated from soil sampled near the Borok settlement in the Yaroslavl Region, Russia, in 2002, and cultivated in the Pratt medium (KNO_3_–100 mg l^−1^; K_2_HPO_4_–10 mg l^−1^; MgSO_4_·7H_2_O –10 mg l^−1^; FeCl_3_·6H_2_O –1 mg l^−1^) with addition of *Pseudomonas fluorescens* bacteria as food. The clone Colp-7 was stored in the collection of live protozoan cultures at IBIW RAS, but perished after two month of cultivation.

Freshwater culture of kinetoplastid *Parabodo caudatus* (Dujardin 1841) Moreira, Lopez-Garcia et Vickerman 2004 was used as food for clones Colp-7a, Colp-14, and Colp-62. *Parabodo caudatus* (strain BAS-1, IBIW RAS) was isolated from Shoensee lake, Ploen, Germany in November 2001 and cultivated in the Pratt medium with addition of *Pseudomonas fluorescens* bacteria as food. The clone Colp-7a is stored in the collection of live protozoan cultures at IBIW RAS.

Marine culture of kinetoplastid *Procryptobia sorokini* (Zhukov 1975) Frolov, Karpov and Mylnikov 2001 was used as food for the clone Colp-5. *Procryptobia sorokini* (strain B-69, IBIW RAS) was isolated from coastal samples collected from the White Sea (salinity 12‰) near the Marine Biological Station of the Zoological Institute, Russian Academy of Sciences (Kartesh) in May, 1986 and cultivated in the marine Schmalz-Pratt’s medium (NaCl–28.15 g l^−1^, KCl–0.67 g l^−1^, MgCl_2_·6H_2_O–5.51 g l^−1^, MgSO_4_·7H_2_O–6.92 g l^−1^, CaCl_2_·H2O–1.45 g l^−1^, KNO_3_–0.1 g l^−1^, K_2_HPO_4_·3H_2_O–0.01 g l^−1^ with a final salinity of 35‰) with addition of *Pseudomonas fluorescens* bacteria as food. The clone Colp-5 was stored in the collection of live protozoan cultures at IBIW RAS, but perished after one month of cultivation.

### Microscopy

Light microscopy observations were conducted using the Carl Zeiss AxioScope A.1 and Biolam-I (Russia) microscopes equipped with DIC and phase contrast water immersion objectives (63x and 70x). The microscopes were equipped with the analog video camera AVT HORN MC-1009/S connected to the Panasonic NV-HS 850 video recorder. Images were acquired using the VHS and S-VHS technology and subsequently digitalized. Short video sequences were obtained in order to facilitate cell identification and describe cell movement, reproduction and feeding behavior. Cells were centrifuged (5000×*g* for 15 minutes, room temperature) and fixed by a 2% solution of OsO_4_ and 0.6% glutaraldehyde (0.05 M cacodylate buffer) for 15–30 min at +1°C prior to electron microscopy analysis. Fixed cells were dehydrated in ethanol and acetone and embedded in Epon-Araldite resin. Pictures were taken using the JEM-1011 transmission electron microscope. The same fixed cells were dried in Critical Point Drying Apparatus and observed using JSM-6510LV scanning electron microscope.

### Gene Sequencing and Phylogenetic Analyses

Cells grown in clonal laboratory cultures were harvested following peak abundance after eating most of the prey. Cells were collected by centrifugation (10000×*g* for 10 minutes, room temperature). Genomic DNA was extracted from fresh cells of Colp-14 and Colp-62 using the Epicentre DNA extraction kit (Cat. No. MC85200). 18S rRNA gene was amplified using general eukaryotic primers (PF1: GCGCTACCTGGTTGATCCTGCC and FAD4: TGATCCTTCTGCAGGTTCACCTAC), cloned, and sequenced. Sequences of *C. edaphicum*, Colp-5, Colp-7 and Colp-7a were amplified as described previously [36]. 18S and 28S rDNA from seven colponemid isolates were aligned to 66 rDNA operon sequences from diverse eukaryotes including a representative set of alveolates, stramenopiles and rhizarians. Sequences were aligned using the local-pair algorithm in MAFFT 6.857b [59] and trimmed in Gblocks 0.91b [60] using b1 = 50%+1, b2 = 50%+1, b3 = 12, b4 = 4, b5 = h parameters. The resulting phylogenetic matrices had 1562 sites (18S rDNA) and 3942 sites (rDNA operon). Phylogenetic analyses were conducted in RAxML 7.28 [61] using -m GTRGAMMA -f a -# 1000 parameters, PhyML 3.0.1 [62] using -m GTR -t e -f e -v e -c 8 -a e -b -4 -s BEST –n_rand_starts 20 parameters, and MrBayes 3.2.0 [63] using lset nst = 6 rates = invgamma ngammacat = 4 parameters, 2 chains and 25% burnin after 5000000 generations. Approximately unbiased test of an alternative tree topology in which *Acavanema* and *Colponema* were placed as sisters was calculated in Consel v. 1.20 based on RAxML per-site log likelihood values for the 18S+28S rDNA dataset.

### Nucleotide Sequence Accession Numbers

Sequences of Colp-14 and Colp-62 were deposited in GenBank under accession numbers KJ598080 and KJ598081.

### Nomenclatural Acts

The electronic edition of this article conforms to the requirements of the amended International Code of Zoological Nomenclature, and hence the new names contained herein are available under that Code from the electronic edition of this article. This published work and the nomenclatural acts it contains have been registered in ZooBank, the online registration system for the ICZN. The ZooBank LSIDs (Life Science Identifiers) can be resolved and the associated information viewed through any standard web browser by appending the LSID to the prefix “http://zoobank.org/”. The LSID for this publication is: urn:lsid:zoobank.org:pub: EE79BEE5-DECC-45BD-94B0-44C0305EC83B. The electronic edition of this work was published in a journal with an ISSN, and has been archived and is available from the following digital repositories: PubMed Central, LOCKSS.

## Results

### Morphology, Movement, and Feeding of Novel Organisms

**Figure 1:**
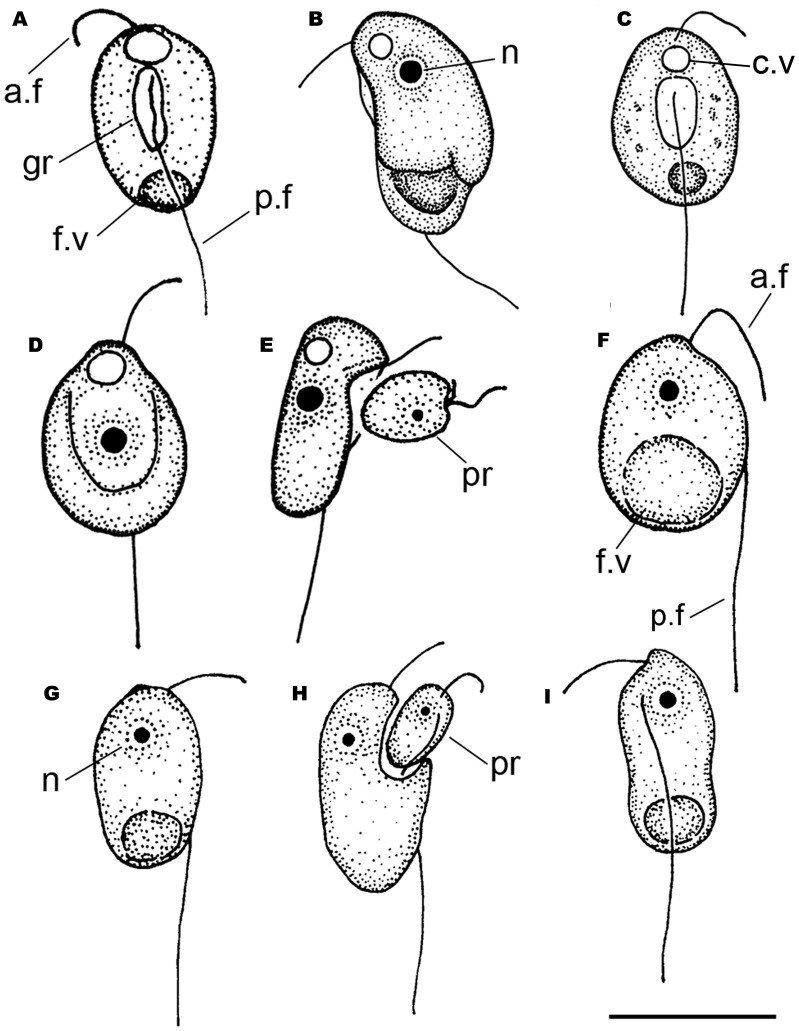
Drawings of *Colponema vientamica* (a–e) and *Acavomonas peruviana* (f–i): (a, c) general view, short ventral groove, large food and contractile vacuoles; b) bean-shaped cell, nucleus, large food vacuole; d) ovoid cell with wide ventral groove; e) feeding of a starved cell with small anterior rostrum; (f, g, i) general view, large food vacuole and nucleus; h) feeding on the prey. a.f – anterior flagellum, c.v – contractile vacuole, f.v – food vacuole, gr – longitudinal groove, n – nucleus, p.f – posterior flagellum, pr – prey. Scale: 10 µm for all figures.

*Colponema vietnamica* sp. n. (clones Colp-7 and Colp-7a).–Young cells are elongated-oval, rigid, not flattened, 10.5–14.5 µm long and 5.0–6.5 µm wide with a small anterior rostrum and a rounded posterior end (Fig. 1a–e, 2a–k). The anterior end of the cell is usually wider than the posterior one. Two heterodynamic flagella originate from a relatively short and weakly pronounced ventral groove near the anterior cell end. The anterior flagellum is about half of the cell length, makes flapping movements, and often curves to dorsal cell surface. The posterior flagellum is about 1.5 times the cell length and sometimes undulates in the longitudinal ventral groove. A large contractile vacuole is located at the anterior end. A spherical nucleus is situated in the center of the cell or slightly closer to the anterior end. The cytoplasm contains light-refracting granules.

**Figure 2:**
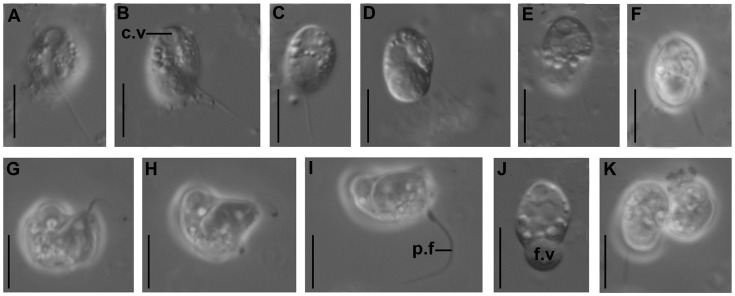
Light micrographs of *Colponema vientamica*. c.v – contractile vacuole, p.f – posterior flagellum, f.v – food vacuole. Scales: 10 µm for all figures.

Cells of Colp-7 and Colp-7a strains swim rapidly in a spiral or zigzag, usually near the bottom of Petri dishes. The organism is an obligate predator; it takes up smaller flagellates and quickly perishes in the absence of prey. Feeding on bacteria was not observed. After an initial contact with prey, the *C. vietnamica* cell stops and captures the prey intact in the longitudinal groove within 1–2 minutes (Fig. 1e, 2g, i). *Colponema vietnamica* actively fed on small *Spumella*-like chrysomonads in natural samples and both *Spumella* sp. (strain OF-40) and *Parabodo caudatus* (strain BAS-1) in the culture. In the natural samples, the predator sometimes attacked a larger prey, e.g., *Thaumatomonas* sp., but did not succeed in capturing it. Cannibalism was not observed. A large food vacuole is formed at the posterior end of cell-body following the feeding (Fig. 1a, b; 2d, f, j). As a result of that, *C. vietnamica* cells become wider (8.5–9.0 µm), bean-shaped or ovoid; the anterior rostrum and longitudinal groove are not visible at this stage. Flagellates begin to reproduce after consumption of several prey cells. Cells multiply by binary longitudinal division (Fig. 2k). Reproduction or resting cysts have not been found in culture. Feeding and reproduction are rapid, so that in general the predator has eaten all prey cells in the Petri dish within 2–3 days (the initial concentration of OF-40 and BAS-1 prey was about 2×10^6^ individuals ml^−1^).

*Acavomonas peruviana* n. gen. n. sp. (clone Colp-5).–Cells are elongated-oval or egg-shaped, rigid, not flattened, 9.5–13.5 µm long and 6.0–10.0 µm wide, with a small anterior rostrum and rounded posterior end (Fig. 1f–i, 3a–n). Two flagella originate near the anterior cell end. The anterior flagellum is about the cell length, very thin, makes flapping movements, and often invisible because it frequently curves to the dorsal cell surface. The posterior flagellum is about 2 times the cell length, straight and rigid, and usually does not move vividly. The nucleus is spherical and situated in the anterior end of the cell. A large food vacuole is located at the posterior end of cell-body (1F, G, I; 3A, B, G). The cytoplasm contains light-refracting granules. No contractile vacuole was found (Colp-5 is a marine species).

**Figure 3:**
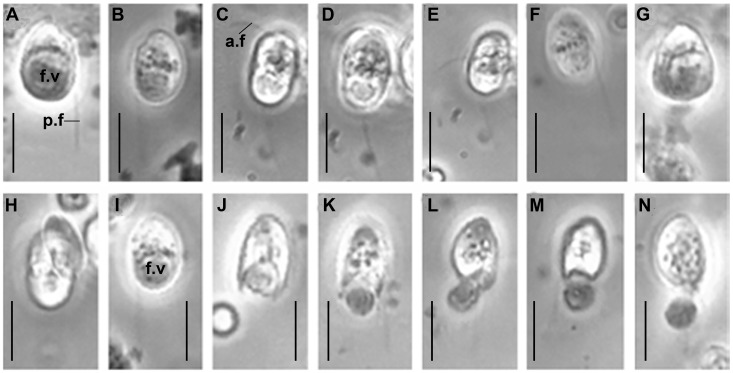
Light micrographs of *Acavomonas peruviana*. c.v – contractile vacuole, a.f – anterior flagellum, p.f – posterior flagellum, f.v – food vacuole. Scales: 10 µm for all figures.

Cells of the *A. peruviana* Colp-5 strain swim rapidly, rectilinear or in a spiral, usually near the bottom of Petri dishes. Sometimes cells swim slowly in a wide curve and smoothly rotate 180°. The organism is an obligate predator and consumes smaller flagellates, quickly perishes in the absence of prey. *Acavomonas peruviana* captures the intact prey; no specialized structures for feeding (longitudinal groove or cytostome) were observed. The organism actively fed on small kinetoplastid *Procryptobia sorokini* in culture (Fig. 1h). *Acavomonas peruviana* sometimes attacked larger prey like cryptophytes in natural samples (Fig. 3h), but did not succeed in capturing it. Cannibalism or feeding on bacteria were not observed. A large food vacuole is formed at the posterior end of the cell following the feeding; the small anterior rostrum is usually not visible at this stage. Undigested remains of the food vacuole are ejected as a round particle from the posterior end of the cell upon completion of digestion (Fig. 3i–n). This process of defecation is very fast, taking only seconds. Cells multiply by binary longitudinal division. Reproduction cysts or resting cysts were not found.

**Figure 4:**
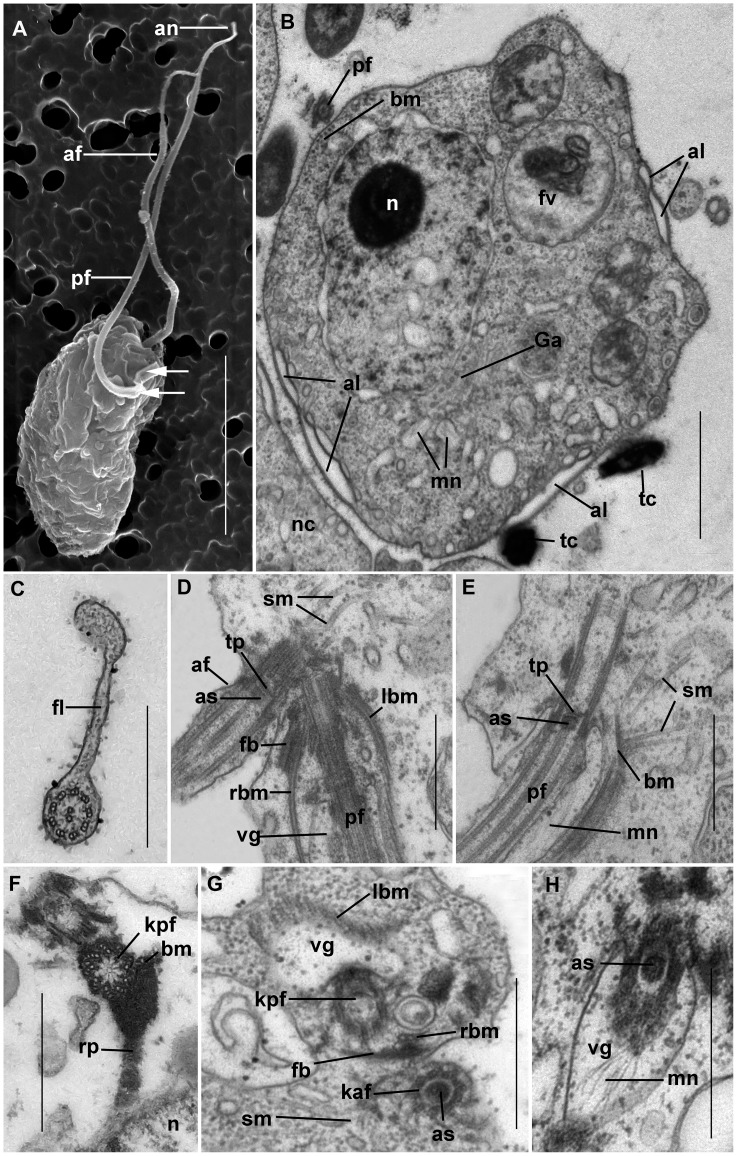
Ultrastructure of *Colponema vietnamica*. a) Electron micrograph of the cell. Anterior and posterior flagella (af and pf) end with narrowing tips – acronemes (an). The arrows point to the short fold of proximal part of the pf. b) The transversal section at the level of the nucleus (n). In some places the cell contains the alveoli (al) beneath the plasmalemma. Small cytosolic vesicles contain the rudiments of the mastigonemes (mn). Golgi apparatus (Ga) is situated close to the nucleus. Discharged toxicysts (tc) are found outside of the cell. The band of 5+1 microbules (bm) accompanies the pf. Food vacuole (fv) contains remnants of a prey cell. The neighboring cell (nc) is seen. c) The cross section of the pf. The thin fold (fl) is visible. d) Flagella are arranged mutually at an angle of 45 degrees. Right and left bands of microtubules (rbm and lbm) and fibrous band (fb) emerge from the bases of the pf which runs inside the short ventral groove (vg). The secondary microtubules (sm) run near af. The axosoma (as) is visible above the transverse plate (tp). e) The pf inside vg. sm originate from the microtubular band (bm). The conspicuous as is located above the transverse plate (tp) of the flagellum. Mastigonemes (mn) cover the pf. f) The short amorphous rhizoplast (rp) extends from the kinetosome of the posterior flagellum (kpf) towards the nucleus (n). G. Kinetosome area. Three microtubules (rbm) and fibrous bands (fb) lie close to the kpf. vg is armored by the left band of microtubules (lbm). Kinetosome of anterior flagellum (kaf) contains an axosome (as). Single secondary microtubules (sm) are seen. h) pf passes in the vg and bears thin mastigonemes (mn). as is visible in the transitional zone of the flagellum. Scales: 0.5 µm in (c–h); 1 µm in b); 5 µm in a).

**Figure 5:**
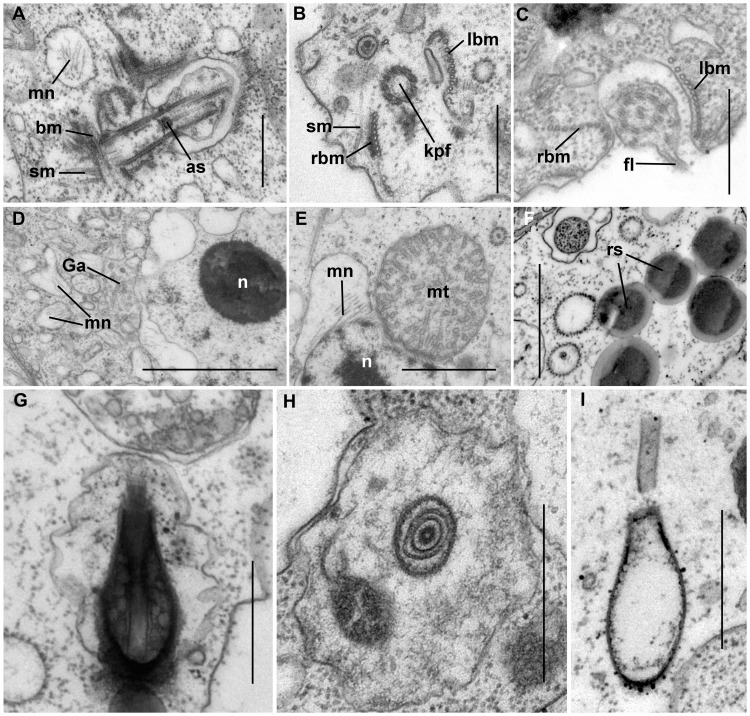
Cell organelles of *Colponema vietnamica*. a) The appearance of secondary microtubules (sm) from the band of microtubules (bm) going near the proximal end of the kinetosome of the flagellum. Mastigonemes (mn) are included inside the vesicles. Axosome (as) of the flagellum resembles a muff. (b, c) The arrangement of the right band of microtubules (rbm) and left band of microtubules (lbm) lying parallel to the posterior flagellum (pf). The single secondary microtubule (sm) is visible. The band of the microtubules surrounds the kinetosome of posterior flagellum (kpf). d) The nucleus (n) contains a conspicuous compact nucleolus. The extensions of the perinuclear space have mastigonemes (mn). Golgi apparatus (Ga) lies close to the nucleus. e) The mitochondria (mt) contain tubular cristae in cross section. Mastigonemes (mn) are formed inside the perinuclear space. f) Osmiophilis granules represent storage compounds (rs). (g, h, i) The structure of the toxicysts. The longitudinal and transversal sections of mature toxicysts g) and h), empty discharged toxicyst i). Scales: 0.5 µm in (a–c), (h–i); 1 µm in d), e), f).

Ultrastructure of *Colponema vietnamica* sp. n. (clone Colp-7a).–The surface morphology of a fixed cell is presented in Fig. 4a. Both flagella end with short, narrowed tips – the acronemes (Fig. 4a). The posterior flagellum bears a short proximal fold (Fig. 4a, the arrows). The cell is surrounded by the plasmalemma. Covering scales are absent. Flattened alveoli are situated just beneath the plasmalemma and together form the cell pellicle (Fig. 4b). Alveoli are often positioned in two adjacent layers and their size and shape vary greatly. Alveoli lack fibrils or plates. Micropores were not observed. The fold of the posterior flagellum is thin and contains fibrous material (Fig. 4c, 5c).

Both flagella have an ordinary structure (9+2) in section (Fig. 4c–d). The flagellar kinetosomes (basal bodies) lie approximately at a 45 degrees angle to each other (Fig. 4d). The transitional plate is situated at the cell surface level (Fig. 4d, e). The remarkable muff-shaped axosoma lies just above this plate (Fig. 4d, e, g, h, 5a). The anterior flagellum is naked, but the posterior one bears thin non-tubular hairs (mastigonemes) (Fig. 4e, h). The mastigonemes are formed inside perinuclear space and can be found in vesicles within the cytoplasm (Fig. 4b, 5a, e). The kinetosomes are relatively long and have a wheel-shaped structure.

The ventral groove starts from the posterior flagellar pocket and extends backwards (Fig. 4d, g, h). The groove is short and supported by two merging microtubular bands. At the anterior end, the right band begins with three microtubules and grows to consists of 8–10 microtubules, and the left band consists of 11–15 microtubules (Fig. 5b, c), but at the level of the nucleus only one band of 5+1 microtubules remains (Fig. 4b). Right and left microtubule bands and a short fibrous band all emerge from the base of the posterior flagellum (Fig. 4d). The kinetosome of the posterior flagellum produces dark-stained (osmiophilic) fibrillar rhizoplast, which extends towards the nucleus (Fig. 4f) and the microtubular bands supporting the ventral groove. The kinetosome of the anterior flagellum initiates a fan-like bunch of the secondary microtubules, which are not organized as a band, although in some sections, the secondary microtubules appear to originate from a microtubular band that runs near the kinetosome (Fig. 4e, 5a). Individual secondary microtubules are also found in the cytoplasm (Fig. 5b).

The vesicular nucleus has a central nucleolus represented by an electronic dense material (Fig. 4b, 5d). Mitochondria are oval and have tubular cristae (Fig. 5e). The Golgi apparatus is positioned close to the nucleus (Fig. 4b, 5d). Osmiophilic, dark-stained granules 0.3–0.5 µm in diameter likely representing storage compounds were observed (Fig. 5f). The extrusive organelles of colponemids are referred to as toxicysts, The exact origin and function of which remain unclear. The toxicysts are enclosed inside a vesicle and situated beneath the plasmalemma and alveoli. Toxicysts are amphora, or bottle shaped (Fig. 5g), are 0.7–0.9 µm in length and 0.3–0.4 µm in width, and consist of a capsule, a cylindrical head, an internal cylinder, and a matrix that contains small, dark, vesicular inclusions (Fig. 5g, h). Intact toxicysts can be found outside the fixed cell. After discharging the toxicyst, an empty capsule and short tube remain in the cytosol (Fig. 5i). Food vacuoles contain remnants of the prey cells (4b). Bacteria were not found in food vacuoles.

### Phylogenetic Analyses

**Figure 6:**
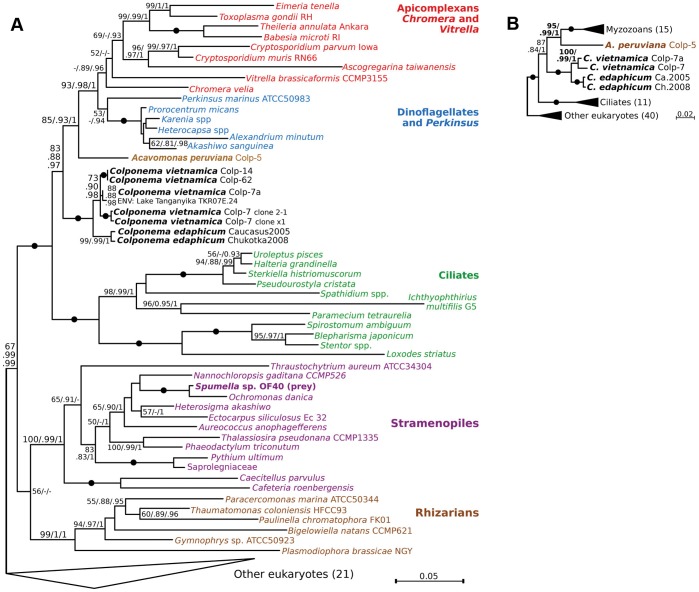
The phylogenetic position of *Colponema* and *Acavomonas* species (in bold). RAxML phylogenies of eukaryotes based on the 18S rDNA dataset (a), and the 18S+28S rDNA dataset (b). RAxML rapid bootstrap, PhyML aLRT and MrBayes posterior probability branch supports are shown at branches (>50/>0.8/>0.9 are shown as significant; dashes indicate insignificant support). Black dots indicate complete support (100/1/1). Numbers of sequences in collapsed clades are shown in brackets.

Recently, we showed that colponemids fell into two distinct groups in the phylogeny of alveolates: Colp-5 branched at the base of the Myzozoa (apicomplexans, dinoflagellates, and their close relatives), while Colp-7 and Colp-7a branched deeper, and were sister to the only described strain with molecular data, *C. edaphicum* [36]. Here we sequenced 18S rRNA gene (18S rDNA) from two new isolates from Vietnam, Colp-14 and Colp-62, and inferred the 18S rDNA phylogeny and combined 18S+28S rDNA phylogeny. Maximum likelihood and bayesian phylogenies including a rich sample of alveolates consistently and robustly placed all colponemid strains within the alveolates, as expected, and outside Myzozoa (Fig. 6). The three major alveolate groups, ciliates, dinoflagellates, and apicomplexans, all formed monophyletic clades, with *Vitrella* and *Chromera* sister to apicomplexans, and *Perkinsus* sister to dinoflagellates, as expected (Fig. 6). Colponemids consistently formed two distinct lineages, one sister to the Myzozoa, and the other in a deeper but unresolved position, as expected [36]. Consistently with previous analyses [36], approximately unbiased test based on the 18S+28S rDNA dataset rejected the direct sister relationship between *Acavomonas* and *Colponema* at the significance level of 0.05 (p = 0.015). The clade containing *C. edaphicum* was further divided into two distinct subgroups, altogether suggesting the strains analyzed here represent three species in two very distantly related genera.

Nearly all the strains (Colp-7, 7a, 14 and 62, as well as *C. edaphicum*) fell into the deeper of the two colponemid clades. The *Colponema vietnamica* morphotype (Colp-7, 7a, 14 and 62) was comparatively diverse at the sequence level, and included an environmental clone from Lake Tanganyika, which grouped with Colp-7a with medium support. This indicates that *Colponema vietnamica* inhabits a similar habitat (tropical freshwater lake) at a similar latitude (8.73° S in Lake Tanganyika vs. 11.29° N in Vietnam) on a different continent, suggesting its distribution is probably more widespread. That *Colponema vietnamica* and *C. edaphicum* formed a distinct and well-supported lineage leads us to conclude that this clade represents the genus *Colponema*.

The single remaining strain (Colp-5, from Peru) was not specifically related to *Colponema* and branched as the sister lineage to Myzozoa with medium to strong support (Fig. 6), consistent with the results of multigene phylogenies [36]. Based on this, we conclude this strain is distinct from the genus *Colponema*, which is consistent with its different morphology (e.g. the lack of a feeding groove), and we therefore place it in a new genus, *Acavomonas*.

## Discussion

### Morphology, Movement and Feeding of *Colponema* and *Acavomonas*

The colponemids are bi-flagellar protists that inhabit both marine and freshwater habitats, and can also be found in the soil [37]–[41]. All *Colponema* species are characterized by rapid swimming using two heterodynamic flagella, a large food vacuole, and an anterior contractile vacuole [37], [38], [42]. Colponemids are obligatory predators, capturing smaller flagellates intact in a longitudinal groove, which is a main distinguishing feature of the genus. Only swimming cells are present in the life cycle; reproduction or resting cysts have not been observed [7], [25], [43].

The ultrastructural features of *Colponema loxodes* Stein 1878, *Colponema* aff. *loxodes* and *Colponema marisrubri* Mylnikov et Tikhonenkov 2009 have been investigated using electron microscopy [7], [25], [26]. All species share a number of distinguishing features at the ultrastructural level. These include the presence of a three-membrane alveolar pellicle lacking alveolar fibrils, theca, or intra-alveolar micropores, a vesicular nucleus with a central nucleolus, two microtubule bands supporting the longitudinal groove, and mitochondria with tubular cristae. The anterior flagellum carries fine nontubular mastigonemes at its proximal end, and the posterior flagellum is characterized by a proximal fold. Small (1 µm) amphora-like or bottle-shaped extrusomes related to the toxicysts described by Mignot and Brugerolle [7] are found in the cytoplasm. They consists of a cylindrical capitulum, tunicate scape with a channel, and matrix, all positioned perpendicular to the pellicle at the proximal end of the cell, close to the cytostomal groove [7], [25], [26].

The new species of *Colponema* described here, *C. vietnamica*, shares the same basic features of the genus, but differs from all currently described species in a variety of morphological characters. Specifically, *C. vietnamica* shares individual characteristics of cell size, cell shape, flagella length, and longitudinal groove length with other *Colponema* species, but collectively possesses a unique suite of these characteristics (see Table 1).

**Table 1:** Comparative morphology of considered organisms.

Species	Cell size, µm	Cell shape	Comparative length ofanterior flagellum inrelation to body length	Comparative length ofposterior flagellum inrelation to body length	Ventral groove	Anteriorrostrum	Contractilevacuole	Nucleusposition
*Colponema* *vietnamica*	10.5–14.5×5.0–6.5(up to 9.0 forsatiated cells)	elongated-oval, not flattened;satiated cells bean-shapedor ovoid	2 times shorter	1.5 times longer	short, weaklypronounced	present	present atthe anteriorend	cell center or closerto the anterior end
*Colponema* *edaphicum*	8–12×2.5–4.5	oval, flattened	about the cell length	2 times longer	short, well-markedat starvingspecimens	present	present atthe anteriorend	anterior or centralcell part
*Colponema* *marisrubri*	8.5–14×4.0–6.5	elongated-oval, not flattened	about the cell length	2 times longer	short, pronounced	absent	absent	anterior cell part
*Colponema* *loxodes*	17–30×8–15	ovoid or bean-shaped,not flattened	about the cell length	1.5 times longer	Long, pronounced	present	present atthe anteriorend	anterior cell part
*Colponema* *globosum*	15×13–14	wide-oval, flattened	slightly shorter than cell	2 times longer	long, deep, withwide curvilinearmargins	absent	present atthe anteriorend	no data
*Colponema* *symmetricum*	9–15	elliptical with widely roundedends, flattened	1.5–2 times longer	3–4 times longer	long, well-visible	absent	no data	slightly below thecell center
*Acavomonas* *peruviana*	9.5–13.5	elongated-oval or egg-shaped,not flattened	about the cell length	2 times longer	Absent	present	absent	anterior cell part

The new genus described here, *Acavomonas*, is morphologically and behaviorally similar to *Colponema*, but also bears important differences. *Acavomonas peruviana* is superficially similar to *Colponema* in that both are small, bi-flagellar, rapidly swimming predatory protist with a very large food vacuole at the posterior end and without cyst or resting stages. However, *Acavomonas* lacks the main distinctive feature of *Colponema*, the longitudinal ventral feeding groove with undulating posterior flagellum, as well as other smaller differences (Table 1). Unfortunately there is no ultrastructure of *Acavomonas* to say whether it also lacks the microtubular bands that support the feeding groove.

The *Colponema* body plan resembles that of excavates. Cells are characterized by a ventral groove and tubular mitochondrial cristae. The posterior flagellum of *Colponema* possesses a fold like in many excavates (e.g. *Histiona*, *Reclinomonas*, *Jakoba*, *Psalteriomonas*, *Trimastix*) [44]–[47]. The architecture of the basal bodies and two ventral roots of *Colponema* recalls those of the retortamonads and jakobids [48]. We have found that *Colponema vietnamica* and *C.* aff. *loxodes* have the same flagellar roots R1 and R2 as are present in typical excavates, *Histiona*, *Reclinomonas*, *Jakoba*, and *Percolomonas* [47], as well root R3 which produce the fan of superficial microtubules armoring the anterior cell end. The ultrastructure of microtubule organizing centers associated with the feeding apparatus in excavates, stramenopiles, apusozoans, amoebozoans, collodictyonids, haptophytes and cryptophytes are all similar in configuration to the microtubular roots supporting the ventral feeding groove in *Colponema* [49], which probably represents the ancestral state in the ancestor of all eukaryotes.

### Possible Ecological Roles of *Colponema* and *Acavomonas*

We observed rapid feeding behavior of *Colponema vietnamica* on *Spumella* sp. and *Parabodo caudatus* and *Acavomonas peruviana* on *Procryptobia sorokini* associated with fast reproduction. Feeding on larger eukaryotes (e.g. *Thaumatomonas* sp.) and bacteria was absent. The findings are consistent with feeding preferences of other colponemid species, and the presence of prey-immobilizing toxicysts throughout this group. These observations support the notion that colponemids and *Acavomonas* are obligate eukaryovores able to rapidly affect the abundance of smaller flagellates, such as ubiquitous heterotrophic chrysophytes and bodonids, in their surroundings. The significance of this behavior in the environment is unknown, but points to a potentially important ecological role in which colponemids and *Acavomonas* participate in regulation of small flagellate abundance, which are themselves often ecologically important grazers of bacteria. Their lifestyle of voracious predators is also consistent with the fact that they have never been observed in great numbers in natural samples. Altogether, the feeding behaviour of *Colponema* and *Acavomonas*, combined with their occurrence and abundance in nature, suggest that they are ubiquitous, but relatively rare, and perhaps only temporarily abundant in favorable conditions. We hypothesize that they play a significant role as mediators of a rapid turnover of small flagellates predominantly in freshwater environments, but perhaps also other major ecosystems (e.g. marine).

### Taxonomic Implications of *Colponema* and *Acavomonas*: Establishment of the New Phyla Colponemidia nom. nov. and Acavomonidia nom. nov

The phylogenetic positions of *Colponema* and *Acavomonas* demonstrated here, and previously in a multigene analysis [36] consistently show they fall into two distinct lineages. The direct sister relationship between both lineages is statistically rejected by the Approximately unbiased test. *Acavomonas* is the closest sister to myzozoans, whereas *Colponema* branches somewhat deeper in alveolates. This tree topology is also consistent with the morphological features of the cells: *Colponema* contains typical alveolate features (cortical alveoli and tubular mitochondrial cristae), but is quite different from *Acavomonas*, which feeds without a feeding groove (which distinguishes it from *Colponema*) by phagocytosis instead of myzocytosis (which distinguishes it from basal myzozoans). The independent branching positions and unique morphologies of both *Colponema* and *Acavomonas* strongly suggests they are independent of all other alveolate lineages, which are currently classified at the level of phyla [24] or eukaryotes of second taxonomic rank according the classification proposed by Adl et al. [50]. Accordingly, we also propose a high-level revision of alveolate taxonomy to accommodate this newly recognized diversity, the Colponemidia to accommodate the genus *Colponema* and its potential close relatives, and the Acavomonidia to accommodate the genus *Acavomonas* and its close relatives.

### Taxonomic Diagnoses

Taxonomic diagnoses of the newly described *Colponema vietnamica* and *Acavomonas peruviana* are given below. We also review the five previously described species of *Colponema*, which were originally described in older literature or in Russian, in order to make these descriptions more accessible.

*Colponema vietnamica* sp. n. Tikhonenkov, Mylnikov et Keeling 2013 (Fig. 1a–e, 2a–k).

Assignment. Eukaryota; Alveolata; Colponemidia.

urn:lsid:zoobank.org:act:EBDBD217-D233-4F2C-A621-00513F1B397B.

Type strain: Colp-7a; sediments of the shallow pool, Bau Sau wetland complex, Cát Tiên National park, Dong Nai Province, S.R. Vietnam. The Colp-7a clone is stored in the collection of live protozoan cultures at IBIW RAS.

Type Figure: 2a.

Description. Cells elongated-oval, rigid, not flattened, 10.5–14.5 µm long and 5.0–6.5 µm wide with a small anterior rostrum and rounded posterior end. Anterior end of the cell usually wider than posterior one. Ventral groove is comparatively short and weakly pronounced. Anterior flagellum about half the cell length and makes flapping movements and often curves to dorsal cell surface. Posterior flagellum about 1.5 times the cell length and sometimes undulates in the longitudinal ventral groove. Large contractile vacuole located at the anterior end. Spherical nucleus situated in the center of the cell or slightly closer to the anterior end. Cells swim rapidly, spiral or zigzag. Organism is an obligate predator; it takes up smaller flagellates (e.g. *Spumella*-like chrysomonads) intact in the longitudinal groove zone. Large food vacuole forms at the posterior end of cell-body following the feeding. As a result of that, cells become wider (8.5–9.0 µm), bean-shaped or ovoid; anterior rostrum and longitudinal groove are not visible at this stage. Cells multiply by binary longitudinal division. Reproduction or resting cysts were not found in the culture.

Comparison. From all other species of *Colponema* studied organism is distinguished by very short anterior flagellum.

Type locality. Sediments contained organic detritus, plant debris and filamentous algae of the shallow pools and lakes of the Bau Sau wetland complex, Cát Tiên National Park, Dong Nai Province, S.R. Vietnam.

Type sequence. Partial small subunit ribosomal RNA gene of *C. vietnamica*: KF651082.

Etimology. The species name means “Vietnam-dwelling”.

**Figure 7:**
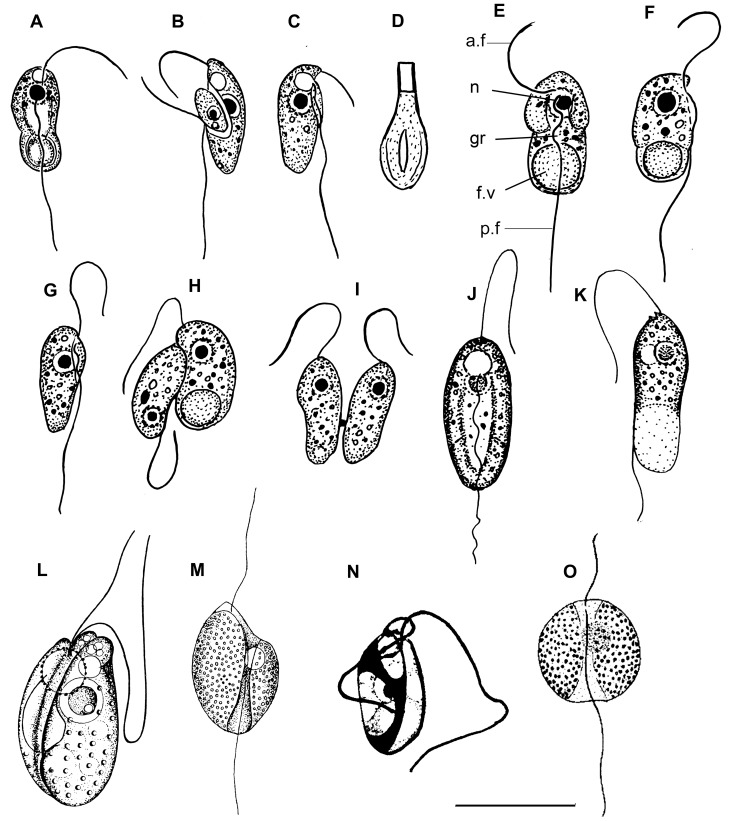
Drawings of colponemids. (a–d) *Colponema edaphicum* (from Mylnikov and Tikhonenkov [43]), (e–i) *C. marisrubri* (from Mylnikov and Tikhonenkov [25]), (j–m) *C. loxodes* ((j, k) from Zhukov and Mylnikov [41]; l) from Chadefaud [42]; m) from Lemmermann [38], n) *C. symmetricum* (from Sandon [39]), o) *C. globosum* (from De Faria et al. [51]). a.f – anterior flagellum, f.v – food vacuole, gr – longitudinal groove, n – nucleus, p.f – posterior flagellum. Scales: (a–c), (e–i) –10 µm; d) –1; (j–m) –20 µm; (n, o) –15 µm.

**Figure 8:**
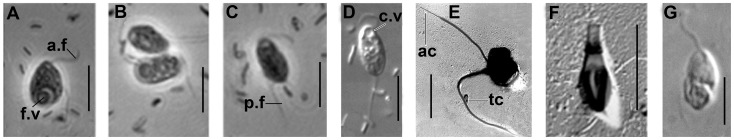
Light and electron micrographs of colponemids. (a–f) *Colponema edaphicum*: a) large food vacuole is visible, b) cell division, (c, d) two heterodymanic flagella and (e, f) extrusive organelle toxicyst (TEM), ((a–c), f) from Tikhonenkov et al. [64], (d, e) from Mylnikov et al. [65]; g) *Colponema marisrubri* (from Mylnikov and Tikhonenkov [25]). a.f – anterior flagellum, f.v – food vacuole, p.f – posterior flagellum, c.v – contractile vacuole, ac – acronema, tc – toxicyst. Scales: (a–e), g) –10 µm; f) –1 µm.

*Colponema edaphicum* Mylnikov et Tikhonenkov 2007 (Fig. 7f–i, 8a–f).

Cell flattened and oval, 8–12 µm long and 2.5–4.5 µm wide with small rostrum and pointed distal part. Flagellar bases situated in anterior part of the cell, subapically. Anterior flagellum about the cell length or slightly longer, often curves to the dorsal cell surface. Posterior flagellum about twice cell length, situated in ventral groove and makes undulating movements there. Large contractile vacuole situated in the anterior part of the cell; median nucleus in the anterior or central cell part. Young specimens with the small rostral anterior end of cell and rounded posterior end (Fig. 7g). Longitudinal groove of starveling specimens are well-marked, and the posterior end of their cells pointed and does not contain food vacuoles. Sometimes this point skewed and displaced on the left side regarding vertical axis of cell. Cell swims rapid, directly or zigzag. The organism is an obligate predator and takes up small bodonids. After feeding, cell forms large food vacuole in the posterior end of cell-body, which becomes roundish. Reproduction or resting cysts have not been found in culture. Cells multiply by binary longitudinal division. Flagellate has extrusomes related to toxicysts (Fig. 7i, 8e, f) about 1 µm length. These extrusive organelles retain their amphora-shape after discharge. The organism is similar to the type-species, *Colponema loxodes* [7]. *Colponema edaphicum* differs from *C. loxodes* by smaller cell body size, shorter anterior flagellum, and elongate cell shape [41], [42]. It is distinguished from *C. globosum* and *C. symmetrica* by the narrower shape of the cell and smaller cell size.

*Colponema marisrubri* Mylnikov et Tikhonenkov 2009 (Fig. 7a–e, 8g).

Cell elongated-oval, 8.5–14 µm long and 4.0–6.5 µm wide. Anterior end of the cell wider than posterior one. Two heterodynamic flagella appear separately from two flagellar pockets. Anterior flagellum about the cell length, posterior one about twice the cell length, situated in short ventral groove and makes undulating movements there. The nucleus is situated anterior, whereas the large food vacuole is posterior. Alveoli are not numerous. The extrusive organelles are of the toxicysts type. Contractile vacuole is absent. Cells multiply by binary longitudinal division. Cysts have not been found. Cells swim in zigzag path near substrate. The organism is an obligate predator and takes up small heterotrophic flagellates (like *Procryptobia sorokini*). Bacterial feeding has not been observed. The organism is similar to freshwater *Colponema loxodes* and soil *C. edaphicum* by shape of the cell and distinguished by the absence of contractile vacuole, short longitudinal groove, and smaller cell size.

*Colponema loxodes* Stein 1878 (Fig. 7j–m).

Cells are ovoid or bean-shaped, 17–30 µm long and 8–15 µm wide, with well-visible rostrum. Anterior flagellum about as long as the cell, and makes flapping movements; the posterior flagellum is about 1.5 times as long as the cell, and undulates in the longitudinal ventral groove. Large spherical nucleus and contractile vacuole situated in anterior part of cell. Large food vacuole in posterior. Cytoplasm contains light-refracting granules. Amphora-shaped toxicysts about 0.9–1.5 µm long. Cells multiply by longitudinal division. Cysts not found. Inhabit fresh-water, including benthos of ponds, reservoirs, sewage waters as well as soils rich in humus. Swims in a spiral pattern, and after making contact with prey stops and within 1–2 minutes captures it. Paralyzing effects on prey not revealed. Pattern of absorption of food was described by Zhukov and Mylnikov [41]. Predators become small and perish in the absence of food. Posterior end of starving individuals pointed, cytoplasm becomes more homogeneous. Flagellates start division after consumption of several prey cells. The species was observed in the coastal zone of the Rybinsk reservoir and in greenhouse soil of Borok settlement [41].

*Colponema globosum* De Faria, Cunha et Pinto 1922 (Fig. 7n).

Cells oval, wide and flattened, with the anterior part wider than posterior one. Deep longitudinal ventral groove, with wide curvilinear margins. Ventral groove narrowed in middle part of cell and widened at the cell ends (especially near the posterior end). Cells are about 15 µm long and 13–14 µm wide. Flagella originating from ventral groove near the anterior end. Anterior flagellum slightly shorter than the cell; posterior flagellum about twice as long as the cell. Median contractile vacuole situated at the anterior end of the cell. Cytoplasm contains light-refracting granules. Cysts not found. Observed rarely in the marine waters of the Rio de Janeiro gulf, Brazil [51].

*Colponema symmetricum* Sandon 1927 (Fig. 7o).

Cells rigid, flattened, elliptical, with widely rounded ends. Distinctive median longitudinal ventral groove divides the cell into two equal parts. Cell length about 9–15 µm. Subapical anterior flagellum is about 1.5–2.0 times as long as the cell; subapical posterior flagellum is about 3–4 times as long as the cell. Spherical nucleus situated slightly posterior to the cell center, near dorsal side and distinguishable only after fixation. Organism always attaches to the substratum by the distal end of posterior flagellum and jerks constantly backwards and forwards. Data about feeding absent. Cysts not found. Rare species, observed in soils of England [39]. The attachment of the cell to the substratum is unusual for representatives of the genus.

*Acavomonas* n. gen.

Assignment. Eukaryota; Alveolata; Acavomonidia.

urn:lsid:zoobank.org:act:D0A71F0F-C760-4236-84D3-E937A0A98C14.

Type Figure: 3a.

Description. The cell is biflagellate, not dorsoventrally compressed, elongated-oval shape with a rounded posterior end. Flagella originate near the anterior cell end. The anterior flagellum makes flapping movements, the posterior flagellum is straight and rigid, does not make obvious movements. A large food vacuole is located at the posterior end of the cell. Longitudinal ventral groove is absent. Obligate fast-swimming predator, consumes smaller flagellates.

Comparison. From very similar representatives of the genus *Colponema* the studied organism is distinguished by the absence of the ventral longitudinal groove.

Type species. *Acavomonas peruviana.*

Etimology. The genus name means “without cave (ventral groove)”.

*Acavomonas peruviana* n. sp. (Fig. 1f–i, 3a–n).

urn:lsid:zoobank.org:act:3B010EA2-C5BE-462B-947F-A2488C6C422C.

Type strain: Colp -5; sediments of saline lake Supay, Pisco Province, Ica Department, Peru. The clone Colp-5 was stored in the collection of live protozoan cultures at IBIW RAS, but perished after one month of cultivation.

Type Figure: 3a.

Description. Cell is elongated-oval or egg-shaped, rigid, not flattened, 9.5–13.5 µm long and 6.0–10.0 µm wide with a small anterior rostrum. The anterior flagellum is about the cell length, very thin, frequently curves to dorsal cell surface. The posterior flagellum is about 2 times the cell length, straight and rigid. A spherical nucleus is situated at the anterior end of the cell. A large food vacuole is located at the posterior end of the cell. Cells swim rapidly, rectilinearly or in a spiral. Organism is an obligate predator; it consumes smaller flagellates (e.g. *Procryptobia*). Reproduction or resting cysts were not found in the culture.

Comparison. Single representative of the genus, from similar species of *Colponema* the studied organism is distinguished by the absence of the ventral longitudinal groove and anterior contractile vacuole.

Type locality. Organic detritus of the saline lake Supay, Pisco Province, Ica Department, Peru.

Type sequence. Partial small subunit ribosomal RNA gene of *A. peruviana*: KF651077.

Etimology. The species name means “Peru dwelling”.

### Two new Alveolate Phyla: Acavomonidia and Colponemidia

Previous alveolate classifications tended to treat the colponemids as unknowns. Cavalier-Smith initially placed *Colponema* in its own class, Colponemea [52], within the infraphylum Protalveolata, phylum Miozoa (replaced by Myzozoa in Cavalier-Smith and Chao [24]). Cavalier-Smith and Chao later emended the infraphylum Protalveolata (within Myzozoa) as a collection of several strange alveolates that were not clearly related to ciliates, apicomplexans or dinoflagellates [24]. This group was retained by Adl et al. [50], despite the fact that many of its members have now been demonstrated to be basal lineages of one of the two major myzozoan lineages (e.g., *Colpodella*, *Chromera*, and *Vitrella* branch with apicomplexans, whereas *Oxyrrhis*, *Perkinsus* and syndinians branch with dinoflagellates), but *Colponema* was removed from Protalveolata and placed *incerta sedis*. In the most recent alveolate re-classification to assign ranks [24], Alveolata were subdivided in to phylum Ciliophora and phylum Myzozoa, with Apicomplexa and Dinozoa at the level of subphyla. In any of these classifications, the phylogenetic positions of *Colponema* and *Acavomonas* found here with rRNA and previously based on a multigene analysis [36] are make them equivalent in rank to ciliates or myzozoans, so here we create two new alveolate phyla to accommodate the newly described diversity that they represent (Table 2).

**Table 2:** Classification of alveolates.

Infrakingdom Alveolata Cavalier-Smith 1991 Primarily single-celled eukaryotes with cortical alveolae, ciliary pit or micropore, tubular or ampulliform mitochondria cristae, extrusomes with dense cores.			
Phylum Myzozoa Cavalier-Smith 2004 Predominantly haploid, typically uninucleate alveolates with zygotic meiosis; lacking separate macronuclei; ancestrally and typically with two centrioles and cilia only; anterior cilium often with simple hairs. Trichocysts typically with a dense basal rod that is square in cross section and a less dense distal region composed of hollow twisted tubules. When trichocysts are present cortical alveoli are typically inflated and morphologically discrete, often with internal plates; when trichocysts are absent they are typically highly compressed and often fused into an inner membrane complex. Myzocystosis and/or rhoptries and micronemes are very widespread, and possibly even ancestral.	Phylum Acavomonidia nom. nov. Free-living bi-flagellar protists with rigid cell and vesicular nucleus. Predators, capture prey as a whole and lack special structures for food uptake (as opposed to sucking structures in Myzozoa employed during myzocytosys). No reproduction or resting cysts.	Phylum Colponemidia nom. nov. Bi-flagellar cells with three-membrane alveolar pellicle, vesicular nucleus has a central nucleolus, two microtubule bands armour the longitudinal groove. The anterior flagellum carries fine nontubular mastigonemes at its proximal end. The posterior flagellum with the proximal fold, undulates in ventral groove. Amphora-like or bottle-shaped extrusomes related to toxicysts type. Micropores are absent. Predators, capturing prey as a whole by a longitudinal groove uptake. Binary longitudinal cell division. No reproduction or resting cysts.	Phylum Ciliophora Doflein 1901 [Ciliata Perty 1852, Infusoria Bütschli 1887] Cells with nuclear dimorphism, including a typically polygenomic macronucleus and at least one diploid micronucleus; somatic kinetids having a postciliary microtubular ribbon arising from triplet 9, a kinetodesmal fibril or striated rootlet homologue arising near triplets 5–8, and a transverse microtubular ribbon arising in the region of triplets 4–6; sexual reproduction, when present, by conjugation typically with mutual exchange of haploid gametic nuclei that fuse to form the synkaryon or zygotic nucleus.
	Class Acavomonea cl. nov. Order Acavomonida ord. nov. Family Acavomonidae fam. nov. urn:lsid:zoobank.org:act:63976BCD-ED40-46E0-8F16-0133DC0896C7 Diagnosis as for phylum Acavomonidia.		
		Class Colponemea Cavalier-Smith 1993. Emend Free-living zooflagellates; centrioles diverge at nearly 90°	
	Type genus *Acavomonas* gen. nov.		
Taxonomy of Myzozoa is not discussed here because we believe that systematics of this phylum cannot be currently resolved with certainty and requires clarification using molecular data.			
		Order Colponemida Cavalier-Smith 1993. Family Colponemidae Cavalier-Smith and Chao 2004 Free-living biciliates with inflated cortical alveoli over all cell surface; cilia subapical; posterior cilium in deep gutter; with toxicysts, but no rhoptries.	
	Type species *Acavomonas peruviana* sp. nov.		
		Phylum is represented now by the single genus *Colponema* Stein 1878	

Phylum Acavomonidia includes the single genus *Acavomonas*. We also established the new family Acavomonidae, order Acavomonida and class Acavomonea to avoid future taxonomical confusion based on the likelihood that other representatives of Acavomonidia with different morphology and molecular phylogenetic topology will be discovered.

We also create the phylum Colponemidia, and move the class Colponemea Cavalier-Smith 1993 and order Colponemida Cavalier-Smith 1993 from Myzozoa to phylum Colponemidia. This classification is strongly supported by molecular phylogenies and by the fact that colponemidians lack apical complex-like structures and do not feed by myzocytosis. However, we retain the order Algovorida Cavalier-Smith 2004 previously classified inside the class Colponemea by Cavalier-Smith and Chao [24] within the phylum Myzozoa, infraphylum Protoalveolata. Algivorids are characterize by apical complex-like structures (roptries and pseudoconoid) and feed by myzocytosis and hence should probably be classified in the class Myzomonadea Cavalier-Smith 2004 (Myzozoa, Protoalveolata) which includes myzocytosic sucking flagellates with similar morphology (*Voromonas* and *Alphamonas*). In the absence of molecular data from Algivorida, however, we suggest to keep Algivorida as an order *incertae sedis* inside Protoalveolata. We also emend the class Colponemea Cavalier-Smith 1993 because it contains the single order Colponemida in the system proposed here (i.e., excluding Algivorida). We improve diagnosis of this class to “Free-living zooflagellates; centrioles diverge at nearly 90°” since the available ultrastructural data [7], [26] show that *Colponema* centrioles do not diverge at “nearly 180°” as stated by Cavalier-Smith and Chao [24].
